# Supplementary material for: No preconscious attentional bias towards itch in healthy individuals
Source: PLoS One. 2022 Sep 2;17(9):e0273581. doi: 10.1371/journal.pone.0273581 (PMC9439194; doi:10.1371/journal.pone.0273581)
Supplement: S1 Table — (DOCX) [file pone.0273581.s001.docx]

**S1 Table.** Self-report questionnaires of individual characteristics (*n* = 127).

|  | Mean (SD) | Range | Cronbach Alpha |
| --- | --- | --- | --- |
| Item on attentional disengagement from- |  |  |  |
| Itch | 3.6 (1.0) | 1 - 5 | - |
| Pain | 2.7 (1.0) | 1 – 5 | - |
| Fatigue | 3.0 (0.8) | 1 – 5 | - |
| Body vigilance (BVS) | 3.7 (1.6) | 0.3 – 8.1 | 0.79 |
| Body Vigilance – item on Itch | 2.2 (2.2) | 0 – 8.2 | - |
| Body Vigilance – item on Pain | 3.3 (2.5) | 0 – 8.8 | - |
| Itch vigilance and awareness (PVAQ-I) | 26.0 (13.9) | 2 – 75 | 0.91 |
| Itch catastrophizing (PCS-I) | 8.6 (8.0) | 0 – 37 | 0.91 |
| Cognitive intrusion of Itch (ECIP-I) | 7.8 (8.7) | 10 – 41 | 0.95 |
| Neuroticism (EPQ-RSS-n) | 7.3 (4.3) | 0 – 12 | 0.78 |
| Psychological distress (DASS-21) |  |  |  |
| Depression | 0.9 (1,6) | 0 – 7 | 0.75 |
| Anxiety | 0.9 (1.5) | 0 – 9 | 0.67 |
| Stress | 1.6 (2.5) | 0 - 11 | 0.88 |

BVS = Body Vigilance Scale (theoretical range 1 – 10);

PVAQ-I = Pain Vigilance and Awareness Questionnaire -adjusted for itch (0 – 80);

PCS-I = Pain Catastrophizing Scale -adjusted for itch (0 – 52);

ECIP-I = Experience of Cognitive Intrusions of Pain Scale -adjusted for itch (10 – 60);

*Note*. Measured on a scale from 1-6 instead of 0-6 like in the original ECIP

EPQ-RSS-n = Neuroticism Scale of Eysenck Personality Questionnaire – revised short form (0 – 12)

DASS-21 = Depression, Anxiety, and Stress Scale- short form (0 – 10 for each subscale)
