# Supplementary material for: No preconscious attentional bias towards itch in healthy individuals
Source: PLoS One. 2022 Sep 2;17(9):e0273581. doi: 10.1371/journal.pone.0273581 (PMC9439194; doi:10.1371/journal.pone.0273581)
Supplement: S2 Table — (DOCX) [file pone.0273581.s002.docx]

**S2 Table.** Spearman rho (*ρ*) correlations between individual characteristics and the Attentional Bias (AB) Index for itch (*n* = 127).

|  | AB Itch |
| --- | --- |
| Item on attentional disengagement from- |  |
| Itch | -0.16 |
| Pain | 0.05 |
| Fatigue | -0.02 |
| Body vigilance (BVS) | -0.06 |
| Body Vigilance – item on Itch | -0.34 ^*^ |
| Body Vigilance – item on Pain | -0.23 ^*^ |
| Itch vigilance and awareness (PVAQ-I) | -0.20 ^*^ |
| Itch catastrophizing (PCS-I) | 0.02 |
| Cognitive intrusion of Itch (ECIP-I) | 0.01 |
| Neuroticism (EPQ-RSS-n) | -0.03 |
| Flanker Index | 0.07 |
| Switch Cost | 0.02 |

* *p* < 0.05

BVS = Body Vigilance Scale (theoretical range 1 – 10);

PVAQ-I = Pain Vigilance and Awareness Questionnaire -adjusted for itch (0 – 80);

PCS-I = Pain Catastrophizing Scale -adjusted for itch (0 – 52);

ECIP-I = Experience of Cognitive Intrusions of Pain Scale -adjusted for itch (10 – 60);

*Note*. Measured on a scale from 1-6 instead of 0-6 like in the original ECIP

EPQ-RSS-n = Neuroticism Scale of Eysenck Personality Questionnaire – revised short form (0 – 12)
