# Supplementary material for: No preconscious attentional bias towards itch in healthy individuals
Source: PLoS One. 2022 Sep 2;17(9):e0273581. doi: 10.1371/journal.pone.0273581 (PMC9439194; doi:10.1371/journal.pone.0273581)
Supplement: S3 Table — (DOCX) [file pone.0273581.s003.docx]

**S3 Table.** Exploratory analyses of the effect of the Flanker Index and Switch Cost on attentional bias during the subliminal dot-probe task for itch: estimates (*ES*) with standard errors (*SE*), significance level (*p-value*) and 95% Confidence Intervals (95% *CI*) (*n* = 125)

|  |  | *ES* | *SE* | *p*-value | *95% CI* |
| --- | --- | --- | --- | --- | --- |
| Model 2a | (Intercept) | 471.4 | 15.19 | < 0.001 | [441.84, 500.88] |
|  | Accuracy | 19.75 | 1.24 | < 0.001 | [15.36, 24.14] |
|  | Congruency | -3.08 | 2.86 | 0.282 | [-8.68, 2.53] |
|  | Group | -11.75 | 11.95 | 0.328 | [-34.98; 11.49] |
|  | Flanker Index | -0.33 | 0.24 | 0.182 | [-0.80, 0.15] |
|  | Switch Cost | 0.07 | 0.07 | 0.333 | [-0.07, 0.21] |
|  | Congruency * Group | 1.52 | 2.28 | 0.505 | [-2.95, 5.98] |
|  | Flanker Index * Congruency | -0.01 | 0.05 | 0.898 | [-0.10, 0.09] |
|  | Switch Cost * Congruency | 0.001 | 0.01 | 0.922 | [-0.03; 0.03] |
| Model 2b | (Intercept) | 471.5 | 15.15 | < 0.001 | [442.04, 500.94] |
|  | Accuracy | 19.75 | 2.24 | < 0.001 | [15.36, 24.14] |
|  | Congruency | -3.34 | 1.96 | 0.088 | [-7.19, 0.51] |
|  | Group | -11.74 | 11.95 | 0.328 | [-34.98, 11.49] |
|  | Flanker Index | -0.33 | 0.24 | 0.176 | [-0.80, 0.14] |
|  | Switch Cost | 0.07 | 0.07 | 0.334 | [-0.07, 0.21] |
|  | Congruency * Group | 1.52 | 2.28 | 0.506 | [-2.95, 5.98] |
|  | Switch Cost * Congruency | 0.001 | 0.01 | 0.920 | [-0.03, 0.03] |

Note. Model 2a: AIC = 491222.8; Model 2b: AIC = 491216.5
