# Supplementary material for: No preconscious attentional bias towards itch in healthy individuals
Source: PLoS One. 2022 Sep 2;17(9):e0273581. doi: 10.1371/journal.pone.0273581 (PMC9439194; doi:10.1371/journal.pone.0273581)
Supplement: S1 Fig — Inspection of the residual distribution of this QQ-plot to assess model fit and potential bias shows that the fitted models were more accurate for lower values, while being slightly biased upwards for higher values. However, inspecting the QQ-plot of Model 3 (not shown here, but similar to the QQ-plot of Model 1) with more sources of information, even after adding scores of the awareness check and control neutral picture type (skin vs. object), could not reduce the observed small bias for higher values. Note. Because all QQ-plots for all the different models are similar, this one is shown as an example for all described models in this study. (DOCX) [file pone.0273581.s004.docx]

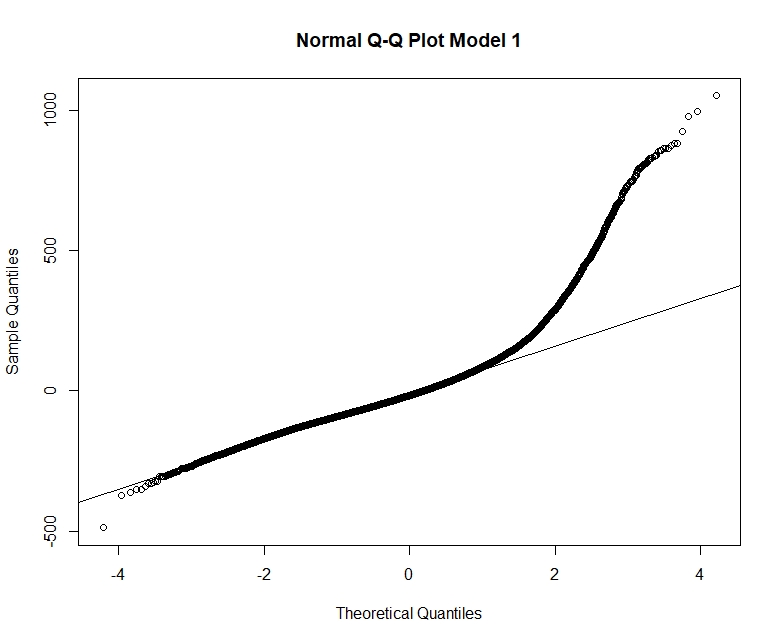


**S1 Fig.** QQ-plot of the residuals of Model 1 for the reaction times outcome of the subliminal dot-probe task for itch. Inspection of the residual distribution of this QQ-plot to assess model fit and potential bias shows that the fitted models were more accurate for lower values, while being slightly biased upwards for higher values. However, inspecting the QQ-plot of Model 3 (not shown here, but similar to the QQ-plot of Model 1) with more sources of information, even after adding scores of the awareness check and control neutral picture type (skin vs. object), could not reduce the observed small bias for higher values. *Note.* Because all QQ-plots for all the different models are similar, this one is shown as an example for all described models in this study.
