# Supplementary material for: No preconscious attentional bias towards itch in healthy individuals
Source: PLoS One. 2022 Sep 2;17(9):e0273581. doi: 10.1371/journal.pone.0273581 (PMC9439194; doi:10.1371/journal.pone.0273581)
Supplement: Attachment — Submitted filename: Response to Reviewer.docx [file pone.0273581.s005.docx]

| To: Dr. Steinborn, section editor of PLOS ONE | | | |
| --- | --- | --- | --- |
|  |  | Date | July 30, 2022 |
| Your reference | Manuscript no. PONE-D-22-05721 | Telephone | +31-71-527 1900 |
| Subject | Response to Reviewer’s Comments | Contact | j.m.becker@fsw.leidenuniv.nl |

Dear Dr. Steinborn, section editor PLOS ONE,

Thank you for considering our manuscript ‘No preconscious attentional bias towards itch in healthy individuals’ for publication in PLOS ONE. A revised version of the manuscript is enclosed.

Below you can find a point-by-point author response to all comments made by the reviewer. Comments are numbered in the order we received them, and answers are preceded by [Reply]. Changes in the manuscript are made with track changes.

We thank the editor and reviewer for their time and constructive feedback. We hope that the implemented adjustments to the manuscript will be satisfactory.

Kind regards on behalf of all co-authors,

Jennifer M. Becker, MSc

**Journal Requirements:**

1. Please ensure that your manuscript meets PLOS ONE's style requirements, including those for file naming.

**[Reply] We have adjusted the formatting of the manuscript to the style requirements of the journal.**

2. Please note that funding information should not appear in the Acknowledgments section or other areas of your manuscript. We will only publish funding information present in the Funding Statement section of the online submission form. Please remove any funding-related text from the manuscript and let us know how you would like to update your Funding Statement.

**[Reply] Thank you for pointing out that the funding statement does not need to be included in the acknowledgement section. The following statement can be included in the online submission form. Thank you very much for adjusting this on our behalf.**

This research is supported by a Leiden University Fonds Fund ([*www.luf.nl*](http://www.luf.nl)) project grant (CWB 7510 / 21‐03‐2O17 / dDM ) and an Innovative Scheme (Veni) grant (451-15-019) of the Netherlands Organization for Scientific Research (NWO, [*www.nwo.nl*](http://www.nwo.nl)), both granted to A.I.M. van Laarhoven. H. Holle was supported by a grant from the Psoriasis Association([*www.psoriasis-association.org.uk*](http://www.psoriasis-association.org.uk)) (award number ST2/18,). The funders had no role in study design, data collection and analysis, decision to publish or preparation of the manuscript.

3. We note that you have stated that you will provide repository information for your data at acceptance. Should your manuscript be accepted for publication, we will hold it until you provide the relevant accession numbers or DOIs necessary to access your data. If you wish to make changes to your Data Availability statement, please describe these changes in your cover letter and we will update your Data Availability statement to reflect the information you provide.

**[Reply] Thank you for pointing out that we need to provide repository information before publication can be completed. According to our institute’s guidelines, we will upload the data to DataverseNL (www.dataverse.nl) and make sure that we provide the link upon acceptance of the manuscript.**

**Reviewer 1:**

Dear reviewer, we are very thankful for the constructive feedback. We really appreciated all the comments and they helped us to improve the manuscript.

1. Data availability statement: You state that some restrictions will apply. What do you mean with this? Please specify.

[Reply] We will upload a publication package, including data and analyses scripts, to a data repository upon acceptance of the manuscript. This publication package can be accessed after permission by the authors and/or our institute’s data managers. In this way data will be available upon reasonable request. We hope that you can agree to this approach.

2. Abstract: Please clarify in the abstract already how itch was induced (what kind of stimuli were used) and how cognitive flexibility and attentional inhibition were operationalized and measured?

[Reply] Thank you for pointing out that more details about the materials would be helpful in the abstract. We now report the requested information in the abstract, see lines 17-18 and 21-22.

3. Has the study protocol been published somewhere? Please give the trial registration number.

[Reply] The study protocol has not been registered or published before. We agree that this would be better practice and have already incorporated this, now also for experimental studies, in our current research practice.

4. Methods:

a. How could it happen that 1 person was tested twice and how did you find out? Was data collected in a pseudonymized manner?

[Reply] Different experimenters were involved during data collection and to keep personal information as confidential as possible full participant details (including names) were not shared between experimenters along the data collection process. Indeed, each participant was assigned a unique participant ID to pseudonymize the data. Only when the researcher merged the pseudonymization file that linked names to participant IDs, it appeared that one person was tested twice. We are very sorry that this has happened due to our effort to keep personal data as confidential as possible. In the manuscript, we have now added that data was collected in a pseudonymized manner in the methods section, lines 99-100.

b. Why were persons stratified for handedness? Please explain why this was necessary to control for. Why were responses given with the index fingers of both hands?

[Reply] Thank you for these questions. Stratification for handedness was done out of precaution. By making sure that left- and right-handed were equally distributed across the conditions, we intended to circumvent any confounding effect by handedness. This way, we excluded the handedness from being a potential explanation for any between-group differences in the study outcomes.

We indeed asked participants to respond with the right index finger to a right button and with the left index finger to a left button. We hoped that this would be most intuitive to the participants and that no delays would occur due to confusion about the correct response side, in regard of the location of the button on the table, and the hand that needed to be used.

c. How much monetary reimbursement was given to the participants? How could this have biased the results? Please consider.

[Reply] Participants received €7,50 for their participation. This is in line with the standard hour rates set by the local Ethical Review Committee that approved the study. This rate is based on compensation for the participants’ time investment. We believe that this rate is not high enough to convince participants to take part in the study. If anything, participants probably have taken part in the study out of curiosity. We do not expect results to be biased by this, since participants were randomly allocated to one of the conditions. We added the compensation participants received to the manuscript, line 121.

d. How was randomization to the itch stimuli and control stimuli conducted? Who conducted randomization?

[Reply] Randomization was done by generating random numbers in Excel (Microsoft Office). This was stratified for gender and handedness to make sure that there would not be only male, right-handed participant in the control condition for instance. We did our best to clarify the randomization in the procedure, lines 112-113.

e. Have the mechanical stimuli been shown to induce itch before? How intense was the itch induced by this method in healthy subjects in former studies?

[Reply] Thank you for drawing attention to this. The same monofilaments were used in two earlier studies (van Laarhoven et al., 2021; Andersen, van Laarhoven et al., 2017) which showed similar descriptive statistics in healthy individuals. However, we have to keep in mind that the surrounding procedures were different and most importantly that in these studies participants were informed that these stimuli would induce itchiness. Therefore, it is possible that expectations about and attention to the stimuli differed and hence, influenced the experience.

f. Why did you come up with new auditory control stimuli and did not use the ones used before?

[Reply] The auditory control stimuli that were used in the earlier study by Swithenbank et al. (2016) appeared to induce some itchiness. The sounds were composed of someone rubbing different body parts (in contrast to scratching for the itch stimuli) which apparently sounded still very similar to scratching. Therefore, we decided to record new control stimuli, that are not at all related to movements on/ manipulations of the skin, but were matched in pitch and length. In this way, we wanted to make sure that the control priming was not associated with itch.

g. Why was it not possible to calculate indicator of total fixation duration for this study?

[Reply] We measured eye-gaze during the presentation of the pictures in the dot-probe task. With our subliminal design, this means that eye-movements were measured during a maximum of 20ms each trial . When we inspected the data and looked at fixation hits and total fixation duration of the two pictures that were presented every trial, we saw that for most trials, the fixation hits and total fixation duration were zero or very close to zero. The distribution of the data around zero made meaningful analysis of the data impossible. Therefore, we recommend using eye-movement measurements in the future only with longer overall presentation times of the material of interest. We admit that we were too simplistic by stating that total duration could not be computed and decided to delete this sentence in the methods section of the manuscript, line 243. Instead, we added to the preprocessing paragraph that also total duration was not analyzable due to excessive zeros in the data set, line 309.

h. Was there a reason to apply this order of filling in the questionnaires? Why were anxiety, depression and strtess measured before the experiment and the other variables not? I assume there was a reason. Please state. Also, I wonder why you applied all these questionnaires? Were there explicit hypotheses regarding all this variables? If yes, please state at the end of the introduction. Otherwise, please state that they were just used in an explorative manner without having specified hypotheses.

[Reply] Thank you for pointing out that this is not yet clear. First, in the current subliminal design, we wanted to make sure that participants are unaware of our major goal to assess preconscious processing of itch-related stimuli. Because all questionnaires, except for the DASS-21, are focusing obviously on itch, we decided to only assess them after attentional bias towards itch had been measured. On the other hand, we wanted to make sure that the attention task would not influence how participants would rate their mental state (depression, anxiety, and stress) and therefore decided to administer the DASS-21 at the start of the study. To clarify that the goal of the study was not explicitly mentioned to the participants, we added a sentence in the procedure, line 107-109. Second, we indeed added the questionnaires for exploratory purposes only. We now mention this in the last paragraph of the introduction, line 85.

i. Page 12, line 260: why do you say mechanical vs. auditory? Does it not have to be mechancial and auditory?

[Reply] Thank you for pointing this out. We have corrected this in the manuscript, line 263.

5. Results

a. There were much more females in the study than males. Please conduct separate analyses for females only in an explorative manner to see whether this changes the results. This would be interesting. Also, please analyse whether woman and men differed in the questionnaire data and induced itch by mechanical and auditory stimuli.

[Reply] There were indeed more female than male participants in our study. However, by stratifying for gender during randomization we made sure that gender was evenly distributed across the priming groups. To confirm that gender indeed did not affect our conclusions, as suggested by the reviewer, we have now conducted a sensitivity analysis testing the main outcome with females only. This sensitivity analysis confirms our initial findings. In addition, we also added gender as a covariate to the model to see whether there would be any effect of gender, again without a significant effect of gender.

Furthermore, as the reviewer requested, we explored whether there were any gender differences concerning the priming and the questionnaire data. However, these must be interpreted cautiously, because the group is highly unbalanced in this case (20 males vs. 105 females) which makes the ANOVA less robust. The priming data showed no significant differences by gender, meaning that females and males rated all stimuli in a similar way. Concerning the questionnaire data, there were some significant findings. Males and females differed significantly in their response on the BVS, the single BVS item for pain, disengagement from pain and itch, all p < 0.05. Still, we believe that this is not confounding our results, because gender was evenly distributed across our priming and control group and all questionnaire effects were controlled for in the model (see Model 3 in the manuscript). We hope that this clarifies your concerns.

b. These are very small differences between itch in the priming group and control group. Were these low differences also observed in former studies? Why did you decide to use only mild stimuli and not stimuli that provoked an increase of at least 2 on a NAS (0-10)? Just because a result is statistically significant, it does not mean that is really meaningful in terms of content. The effect sizes illustrate a small effect. Please consider. Also, what was the itch baseline in both groups? Was this not measured? This would be a real limitation and should be discussed.

[Reply] Thank you for mentioning this discussion point. This study is the first one that compared these specific itch and control stimuli, therefore a comparison to earlier studies is not possible. However, as described above, the itch induction by the mechanical stimuli was in line with previous studies. Nevertheless, we agree that the between-group differences in evoked itch are small, which might be a reason that there was no priming effect in our study. We discuss this limitation and suggest more pronounced stimuli for future studies in the discussion, see lines 488-490.

Indeed, we do not have a baseline measure of itch . The reason for that was, as described earlier, that participants should not be aware that the study was measuring attention to itch or was related to itch at all. In this way, we kept our design preconscious, but indeed cannot guarantee that participants did not have any baseline itch symptoms due to everyday hassles, like mosquito bites (participants with skin conditions were excluded). We agree with the reviewer that this needs to be discussed and we now mention this limitation in the discussion, lines 490-492.

c. Awareness: What does a mean accuracy of .49 mean?

[Reply] Thank you for pointing out that this is not clear yet. During the forced-choice paradigm that was used as an objective awareness check, participants saw pairs of pictures. One of these pictures had been used during the dot-probe task and the other one was completely new to the participants. Because people were obliged to choose one of the pictures during each trial, hence, there is a 50% chance to select the picture that had been presented during the dot-probe task. Accuracy was operationalized as correctly identifying the picture that was used during the dot-probe task, see addition lines 208-209. In the results section, we described that the measured mean accuracy of 0.49 did indeed not significantly differ from chance level, lines 363-364. In this way we support the notion that participants did indeed not consciously process the pictures during the dot-probe task.

6. Discussion

a. Were patients with any itchy conditions (even moskito bites) excluded? Were groups (priming and control group) comparable regarding baseline itch? This comparison is necessary in order to secure that the priming actually worked.

[Reply] Please see our answer to point b. about the results. Due to our subliminal design with deception about the purpose of the study, we did not assess baseline itch, hence were not able to control for this in the current study. Still, we believe that due to the randomization of participants across groups, the influence of potential baseline itch would have been averaged out. Nevertheless, we agree that the lack of baseline itch levels is a limitation to the present study, as we now mention in lines 490-491.

b. Regarding references 27 and 52: Were participants not blinded about the real purposes of the studies and debriefed afterwards?

[Reply] Of course, we want to be very cautious about our interpretation of other studies, but as far as we understood, in these previous studies participants were aware that they participated in itch -related research. This does not mean though, that participants were indeed unblinded for the specific research questions and manipulations – for which there was a debriefing afterwards. In our study, itch was not mentioned at all to the participants, thereby minimizing the influence of itch-related expectations on the outcomes. In the manuscript, we now explicitly mention that participants were kept naïve about any itch-relatedness of the study, lines 107-109 and we elaborated about this point in the discussion, lines 458-461.

c. Please, clearly state that patients with chronic itch and healthy skin controls should differ in their attention towards itch stimuli. I would assume that patients with chronic itch show a (greater) AB towards itch stimuli. What could have prevented the AB in healthy skin controls? Might they have experienced digust? How could disgust moderate AB?

[Reply] Thank you for these interesting and relevant suggestions. We agree that it is likely that patients with chronic itch show a different attentional pattern compared to healthy controls, e.g., in the light of contagious itch also being more intense in patients with chronic itch than healthy controls (see for instance the review by Schut et al., 2015). We rephrased this point in the last sentence of the discussion, lines 506-508.

The possibly influential role of disgust in our findings is a very interesting one. Scratching is often seen as a negative behavior, which one would like to avoid. A potential explanation is that it is considered disgusting, which is understandable from an evolutionary perspective as scratching could signal harmful, infectious agents. So, disgust could have moderated our findings in a way that individuals want to avoid the itchy pictures. Disgust might also be highly related to the stigma that rests on skin diseases, see lines 429-430. Concerning the current stimulus material, it was never investigated whether people experience any disgust related to the pictures (see validation process in Becker et al., 2020). Because there are no skin lesions visible on the material, we would assume that disgust scores are not high. Yet, earlier research has shown that skin-related disgust is an important topic in patients with chronic skin (Lahousen et al., 2016). Therefore, this train of thoughts seems valuable for future research. We added this notion to the discussion, lines433-438.

7. Table S2: What was the item on itch oft he BVS? How do you interprete a negative relationship between the BVS item on pain and the AB Index for itch?

[Reply] The fourth item of the BVS asked the following question: “Rate how much attention you pay to each of the following sensation, using the scale below.” This was followed with several visual analogue scales (sliders) ranging from 0 (none) to 10 (extreme). In addition to the standard items, e.g., heart palpitations or tingling, we added itch and pain as items to this list, see clarification in line 248. Even though there seems to be some consistency in the correlations between the AB index for itch and self-reported attention for itch and pain, in our opinion, interpretation of the few significant correlations should be done cautiously in the light of the high amount of correlation coefficients. A possible explanation might be that a negative AB score actually means avoidance from itch. Therefore, more vigilance towards itch and pain (significant correlations with BVS-itch, BVS-pain and PVAQ-I) might suggest that people who are more aware of bodily sensations might also be more avoidant of potential bodily harm. But this is just speculation at this point. We added this point to our discussion, see lines 481-486.
